# “It’s hard to feel a part of something when you’ve never met people”: defining “learning community” in an online era

**DOI:** 10.1007/s10734-022-00886-w

**Published:** 2022-07-28

**Authors:** Lucy Prodgers, Elizabeth Travis, Madeleine Pownall

**Affiliations:** School of Psychology, University of Leeds, 4 Lifton Place, Leeds, LS2 9JZ UK

**Keywords:** Learning community, Online education, Higher Education, Student support, Sense of belonging

## Abstract

Feeling part of a community of learners has been shown to foster students’ engagement and sense of belonging, leading to higher retention and achievement of learning outcomes. The pivot to online teaching caused by the COVID-19 pandemic has prompted a reappraisal of all aspects of the student experience, including students’ capacity and opportunity to engage in meaningful learning communities online. There has been some emergent literature which considers how to facilitate online learning communities in the emergency remote teaching context prompted by COVID-19. However, there is a notable lack of literature which considers how learning communities are defined, understood, and negotiated by students in this unique teaching context. Given how students’ perceptions of learning communities contributes to Higher Education policy (e.g. through the National Student Survey), this is important to understand. In the present study (*N* = 309), we qualitatively investigated students’ understanding and definition of the term “learning community” during a time of emergency pivot to online teaching and learning. A reflexive thematic analysis of students’ first-hand responses generated three dominant themes: “Feeling connected: Bridging the gap whilst physically distanced”, “Feeling included: Visible and valued”, and “Feeling together: Mutuality and the shared experience”. We discuss the implications for these conceptualisations of an online learning community and suggest ways forward for Higher Education pedagogy.

The COVID-19 pandemic prompted a discipline-wide, global reappraisal of all practices in Higher Education, from student support, to examinations, to teaching delivery. One important part of the student experience that may have been disrupted and changed by COVID-19 is students’ capability for building, participating in, and benefiting from learning communities during their undergraduate education (e.g. see Pownall et al., 2021). There is a plethora of pre-COVID research which has demonstrated how feeling part of a community of learners is useful for fostering student engagement, developing positive staff-student relationships, and improving students’ sense of belonging in Higher Education (Macheski et al., 2018; Moser et al., 2015). The pedagogic benefits of feeling a sense of welcome, belonging, and community are well documented in the literature. For example, feeling part of a community of learners has been positively related to student engagement, student learning, and graduate outcomes (Akyol & Garrison, 2011; Arbaugh, 2008; Krause, 2005; Pike et al., 2011; Zhao & Kuh, 2004). An enhanced learning community culture has also been associated with improved communication, problem solving, and students’ ethical and social sensitivity (Smith & Bath, 2006).

However, as West and Williams (2017) noted, the term “learning community” is contested in Higher Education, with no two groups sharing the same definition. For some, learning community has been defined by a space for understanding, sharing, and facilitating best practice (e.g. Tosey, 2006). Whereas, for others, a learning community is the *act* or process of working collaboratively (Davies et al., 2005). There have been definitions offered in the literature too. Carlen and Jobring (2005, p. 273), for example, noted a learning community is broadly “a learning atmosphere… from which sustainable learning processes are gained through a dialogue and collaborative construction of knowledge”. Others have concentrated on the construction and sustainment of learning communities in Higher Education. For example, learning communities have thought to be promoted and facilitated through various pedagogical and social means, such as “social presence” or “sense of belonging” (e.g. McMillian & Chavis, 1986; West & Williams, 2017), and “teaching presence” (Anderson et al., 2001; Yuan & Kim, 2014). These inconsistencies of definitions are problematic, particularly given how learning community features in Higher Education policy shaping; for example, students in the UK are asked to rate the learning community of their institution in the National Student Survey (NSS; Office for Students, 2021). These ratings serve as a policy instrument that directly informs institutions’ reputation and, more broadly, aims to validly and reliably capture student’s satisfaction in their learning (e.g. see Bell & Brooks, 2017). If items within the NSS are aligned to pedagogic constructs that are ill-defined, or where there are sharp inconsistencies with interpretation, this is problematic for the reliability of NSS as a measurement.

## Learning communities in an online context

To date, the consideration of how learning communities are defined has largely been concerned with face-to-face teaching and learning in Higher Education. Given how the COVID-19 pandemic has prompted a global shift to emergency online learning, it is crucial to understand how the term learning community is understood, conceptualised, and negotiated by students in the current “online era” of Higher Education. As Hodges et al. (2020) have stressed, there is an important distinction to be made between the emergency remote/online teaching prompted by COVID-19, and the kinds of pedagogy that are carefully and intentionally designed to be administered online (such as distance learning courses). The present study is interested in definitions of learning communities online in the context of *emergency* remote teaching and is informed by the vast literature which discusses learning communities in distance learning contexts too.

Some scholars have considered how learning communities are facilitated online (e.g. see Murdock & Williams, 2011). Yuan and Kim (2014), for example, provide a useful set of guidelines for fostering learning communities in online spaces. Yuan and Kim (2014) noted development of a learning community online fostered students’ sense of belonging and connectedness to their environment (Ouzts, 2006), and thus could provide students with a useful opportunity for interaction that is necessary in online teaching and learning. This complements the literature which has provided advice on building effective learning communities online (e.g. Bonk et al., 2004; Dawes & Sams, 2004; Hildreth & Kimble, 2004; Lewis & Allan, 2005; Lowry et al., 2000; McConnell, 2006).

So far in the literature, some scholars have proposed novel ways to promote feelings of learning community in online teaching (e.g. see Abidin et al., 2021). Even in the unique context of COVID-19, there is some emergent literature which considers *how* learning community can be developed online. For example, Safi et al. (2020) described how a “mini-conference” aimed to foster a sense of learning community online during COVID-19. DeKorver et al. (2020) have also provided strategies for facilitating learning communities in physics teaching during the pandemic, and Jamieson (2020) shared mechanisms to preserve learning communities during COVID-19. However, despite this useful literature which demonstrates how to facilitate and build learning communities online, including in the unique context of COVID-19, there is a notable lack of literature which investigates how students *define* the concept of online learning community. Indeed, as already noted, definitions and conceptualisations of learning community as a pedagogic term are lacking in consistency (West & Williams, 2017). We argue that before we can meaningfully create and develop positive learning communities in the online era of COVID-19 education, we must first understand how students define, relate to, and understand what constitutes a learning community online. To achieve this, we qualitatively explored students’ perceptions of the term learning community in an online teaching context.

## Method

### Participants and design

Participants were 309 undergraduate Psychology students enrolled at the University of Leeds, which is a research-intensive UK university. One hundred forty-two (45.69%) participants were in their first year of study, 83 (26.86%) in second year, and 80 (25.89%) were final-year students. Two students were in a year abroad or industry and 2 students did not provide their year. The majority of the sample was female (88.03%), which reflects the population of Psychology students; approximately 80% of undergraduate psychology students are female (Johnson, Madill, et al., 2020; Johnson, Sprowles, et al., 2020), which rises to 85% at Russell Group institutions (the context of the present study). See Table 1 for more demographic detail about the sample of students.

**Table 1:** Demographic breakdown of participants

Demographic groups		Frequency (% total *N*)
Age	Mature students	60 (19.42%)
	Not mature students	247 (90.29%)
	Not given	2 (0.65%)
Gender	Female	272 (88.03%)
	Male	25 (8.09%)
	Non-binary/other	11 (3.56%)
Contact with other students	Regular contact with other psychology students	141 (45.63%)
	No regular contact	167 (54.05%)
Living situation	Living with other students	138 (44.66%)
	Living in halls	100 (38.83%)
	Living at home	63 (20.39%)
	Living alone	7 (2.27%)

Participants were recruited via emails and posts on the Virtual Learning Environment. Ethical approval was granted from the local ethical committee on 20 October 2020 (Reference: PSYC-109). The learning community items reported here were part of a larger questionnaire, which assessed other facets of the student experience, including assessment and feedback practice, online learning preferences, and inclusion. Data were collected during November 2020, at which point students were in the latter part of their first semester of online teaching. During this time, students experienced both asynchronous online teaching (e.g. pre-recorded lectures, instructional videos) as well as synchronous small-groups lessons (e.g. seminars, tutorials, and one-to-one supervisions via online videoing software, such as Microsoft Teams and Zoom).

### Procedure

Participants began by providing demographic information including gender, year of study [first/second/year abroad/year in industry/final year] and whether or not they were a “mature student” (classified as over the age of 21 years old at the start of their studies). We then asked where they were living during the semester [at home/in student halls/in a house with other students/alone] and whether they lived with other psychology students and had any regular contact, including online or in-person, with other psychology students outside of timetabled classes.

Participants were informed the study was interested in the sense of “community” in the School of Psychology. To qualitatively investigate this, students were asked what the term learning community broadly meant to them. For this question, participants were asked to write at least 100 characters. Then, we asked participants to provide an example of when they had particularly felt part of a learning community in the School. Finally, we asked participants to write about ways in which their feeling of being part of a learning community in the School could be improved.

Extracts used in the analysis are identified by year of study, living circumstances and participant number in parentheses (e.g. first year living in Student Halls [60]). The total number of qualitative responses to each question is given in Table 2.

**Table 2:** Number of responses and response rate for each qualitative question

	Responses (*N*)	Response rate (%)
What does the term learning community mean to you?	171	55.34
Please provide an example of when you have particularly felt part of a learning community in the School of Psychology	135	43.69
What would improve your feeling of being part of a learning community in the School of Psychology?	131	42.39
Total responses	437	-

#### Analytic method and approach

Data were analyzed using Braun and Clarke’s Reflexive Thematic Analysis (2006, 2019). This was particularly suitable given the area of interest—students’ definitions and conceptualisations of online learning community—is both under-researched and subjective (Braun & Clarke, 2006). For this reason, we used an inductive approach such that the themes retained a strong link to the data and were not driven by any pre-existing theoretical framework (Braun & Clarke, 2006). This analysis also used a critical realist approach in which the role of language in constructing social realities is recognised, whilst acknowledging these realities are constrained and shaped by our material world (Sims-Schouten et al., 2007). As such, we assert that whilst participants’ understandings of learning community do not uncover a pre-existing “reality” or “truth”, and are socially produced, such understandings are experienced as materially real for the participants themselves.

The data analysis followed the six phases of Reflexive Thematic Analysis (Braun & Clarke, 2006, 2019) and was primarily conducted by the first author. Initially, she actively read the dataset in full and in-depth several times, noting down anything of particular interest, before generating descriptive codes. At this stage responses to the different questions were kept separate, though codes traversed them and were kept broad and semantic, for example, “help and support”, “enabling learning”, and “interaction with teaching staff”. Given the primary interest was respondents’ overall conceptualizations of learning community, once all the data had been coded, the codes and responses for each question were collapsed together. The relationships between codes were then considered and initial themes were generated with a focus on more latent aspects of the data, that is, on the underlying assumptions which shaped or informed the semantic content (Braun & Clarke, 2006). For instance, we considered how “help and support” were conceptualised by respondents, which lead to an initial overarching theme of “feeling supported” underpinned by subthemes such as, “respected, accepted and safe” and “quality interactions”. Themes were then reviewed and refined at both the level of the coded data extracts (reading all of the collated extracts for each theme to check for a coherent pattern) and in relation to the entire data set (by considering whether themes were reflective of the meanings across the data as a whole). Finally, we further refined and defined themes before contextualising them in relation to West and William’s (2017) conceptual framework for learning communities and producing the final report.^1^

Following Braun et al. (2013), the analysis took place in collaboration with the second author who reviewed each stage by reading and re-reading the data, codes and themes as appropriate to ensure coherence and fair representation of the dataset. The third author was involved in reading and discussion throughout.

#### Reflexivity statement

All three authors of this article are Postgraduate Teaching Assistants (PGTAs) working in the School in which the research took place. As PGTAs, we are contracted to teach and contribute to the educational needs of the School alongside our work towards our doctorates. Early in our roles, we were tasked with improving the School’s sense of learning community. We quickly became aware the term itself was ambiguous and wondered how the students themselves oriented towards and conceptualized it. Hence, this research paper was born! We share our unique perspectives below, to contextualize the lens through which this analysis was generated.

##### Lucy Prodgers.

My interests are primarily in critical psychology; as such, my work aims to interrogate the underlying assumptions and values which underpin mainstream psychological discourses. I have been in Higher Education on and off for almost 22 years. I have therefore participated in a varied and diverse range of learning communities both on and offline. I am unsure of when I first became familiar with the term itself, though I feel it is a “slippery” concept. My involvement in and understanding of the various learning communities I have encountered are very much bound to particular times and contexts; thus a simple, stable meaning seems elusive. As a White, female, middle-class, mature and disabled early career researcher, I am aware these experiences will inevitably have been impacted by the particular intersection of my own privileges and disadvantages.

##### Elizabeth Travis.

My academic research interests lie within applied health psychology. Studying for my undergraduate and master’s degree, the lasting relationships, connections and fun had have always been of equal importance to the knowledge and skills gained along the way. I personally need to feel a sense of belonging and part of a community to function well and be happy, and these traits have impacted my perspective and where my interests lie within this project. In my previous career before academia, I spent time leading a colleague engagement team to improve morale, staff development and communications.

##### Madeleine Pownall.

I am a young, White, female, early-career researcher with a strong interest in feminist approaches to teaching and learning. This means I am particularly attuned to issues such as equality, diversity, and inclusion, which shapes the lens through which I view issues of learning community in Higher Education. I am sympathetic of the student experience, as it is so closely tied to my own recent lived experience. This means I have a heightened concern for student support, particularly in areas that mirror my own experiences (e.g. the need to feel heard and supported by academic staff).

## Results

Data for this study can be openly accessed via this Open Science Framework page: https://osf.io/zw94s/. Participants provided consent for their anonymised responses to be made available. Three themes were generated from the data, all of which connected to respondents’ overall experience of being part of an online learning community: *Feeling connected: Bridging the gap whilst physically distanced*; *Feeling included: Visible and valued*; and *Feeling together: Mutuality and the shared experience*. In line with the epistemological underpinnings of the study, we do not focus on the prevalence or the quantity of responses related to each theme but aim to capture aspects which are particularly illuminating in relation to the overall research question (Braun & Clarke, 2006, p. 82).

### Feeling connected: bridging the gap whilst physically distanced

There was a general sense that it was difficult to obtain a feeling of learning community when learning was delivered exclusively online and for some this was not a possibility at all: “with being at home learning, it doesn’t mean much” [First year living in student halls [62]). Students noted being online led to feeling “very distanced and separate” (First year living in student halls [60]) as “you can’t get to know people as well as via face-to-face” (First year living in student halls [99]). For others, it may have exacerbated a sense of isolation that pre-dated the pandemic: “It’s so lonely and anti-social. I’ve hardly made any friends on my course because it’s so difficult to actually interact with people” (Second year living with other students [101]). Indeed, according to West and Williams (2017), absence of loneliness is key to a sense of belonging within “Relationship” learning communities.

Central to this sense of isolation was the inability to physically see, meet and share space with fellow students. In our data, the experience of studying communally and feeling physically part of a group of learners was crucial for some, be this within shared study spaces, such as the library (“In the library when you would see everyone working” (Final year living with other students [226]), “stuck in the clusters/library computer rooms late at night trying to get reports done with other psychology students” (Final year living with other students [240])); formal teaching contexts, such as lectures, seminars and tutorials (“When I could see everyone in lectures” (Second year living with other students [245]), “in my tutorial classes” (First year living at home [31]); or more general and informal spaces on campus, such as the student common room (“If I could come back into campus and be at the student hub in psychology building” (Second year living with other students [237])). Simply “being able to see people face to face” (First year living with other students [268]) and “spend more time around more psychology students” (Final year living with other students [149]) was of great importance, and the lack of opportunity to do so during the pandemic led many to feel a sense of learning community was nigh on impossible: “I wish we could meet in person because that would really help” (First year living in student halls [285]). For those new to the university, such as first year students, this physical barrier was often more apparent given there was no prior opportunity to meet others and make friends: “it’s hard to feel a part of something when you’ve never met people” (First year living in student halls [75]); “being able to meet more people from the school of psychology [would improve a sense of community]” (First year living in student halls [22]).

In the absence of being physically proximal, being able to see, interact and meet with one another virtually on a regular basis was seen to strengthen learning communities online. As one student noted, it is “important to meet fellow students” (First year living with other students [16]) and another indicated frustration when peers did not use cameras or audio during sessions: “I always have my microphone and camera on during lives and I find I am, most of the time, the only one that does. So I don’t feel like I am in a community.” (First year living in student halls [270]). Online social events, such as welcome activities hosted during Fresher’s or Orientation Week (“the welcome week live introduction sessions” (First year living in student halls [32])), and smaller group activities (“Perhaps to have some smaller meet ups/events” (First year living in student halls [25]), “more Virtual Student Meet ups” (Second year living alone [113])) were given as examples of how to achieve this sense of social proximity. These aspects indicate overlap between West and Williams’ (2017) “Access” and “Relationships” communities: whilst quantity and quality of time together were important (Access), reciprocity (Relationships) was also key to achieving this sense of closeness.

Being physically proximal and using shared study spaces when on campus had previously led students to more spontaneous and informal learning opportunities which helped to consolidate and extend their understanding of course content: “I would often discuss with fellow students in library study breaks our understandings of the content and its wider implications. This was not only helpful in consolidating learning but broadening perspectives” (Final year living at home [36]). Such spontaneous interactions were difficult to reproduce in online contexts, though students appreciated online peer group chats via social media sites, such as Facebook. Some felt this was the only way to achieve a sense of learning community online: “[the] Facebook group chat with about 100 psychology students […] is the only effective way of feeling like a community” (First year living in student halls [255]). Having the space to informally discuss issues and work through them together solidified a sense of community, which was perhaps more important when online contexts prevented the spontaneous interactions which often took place during or immediately after in-person teaching sessions.

Online interactions potentially inhibited the ability to get to know and connect with others effectively. As one participant noted, “Having more seminars” enabled students to “properly meet people” (Final year living with other students [09]), signifying that in other online learning contexts connection with others was only superficial. Not all students felt they would benefit from more online group work or smaller group interactions, however. One respondent noted group work “is difficult to organise at the best of times, even worse online” (Final year living at home [269]), whereas another stated “[virtual] break out rooms are the worst things invented” (Second year living with other students [238]). Interestingly, the latter argued the awkwardness associated with online group work was not an issue when delivered “in person”, suggesting it was the online context itself that created this discomfort, whilst the former indicated the online context heightened existing difficulties further.

The stresses and strains associated with the pandemic and the shift to online learning was inevitably overwhelming for many students. Just as with in-person group work, not all responded well to group activities online, which were perceived to be even more socially awkward. Sensitivity to the differing needs of the student body is therefore required.

### Feeling included: visible and valued

Both academic and more pastoral forms of support were commonly identified as key components of a successful learning community. Importantly, it was clear that basic academic support alone was not enough and “more communication and support” was needed “asides (sic) from just lecture content and assignment instructions – a more personal approach” (Second year living with other students [280]). Although access to resources and awareness of what support was available was clearly acknowledged as important (for example, “Having access to information and resources when needed” (Final year living with other students [235]); “knowing which staff I should approach for certain issues” (Second year living with other students [94])), the successful engagement with and use of these resources was underpinned by support being more “personal”, enabling students to feel more at ease and comfortable with peers and staff. This shares links with West and William’s (2007) “Access” and “Relationships” characteristics, demonstrating the value of reciprocal relationships which provide space for knowledge sharing and trust, to forge connections and meaningful ties.

For one respondent, a successful learning community was, “A place of comfort where you are supported” (First year living at home [14]), whereas for another it is related to “Feeling involved and comfortable with other students and teaching staff” (First year living in student halls [82]). As such, students not only needed access to opportunities to communicate, interact and engage with peers, staff, and other university resources, they first had to feel “comfortable” and “involved” enough to do so. As one student noted, “Being able to be open about when there’s something you’re struggling with” (Second year living with other students [67]) is key. Notably, being part of a large cohort made this more important given “it is easy for personal problems to go unnoticed” (Final year living with other students [265]). If students feel invisible, they may be less likely to “reach out for help”.

Staff played a vital role in developing a sense of comfort by being responsive and approachable (“a few teaching staffs (sic) were very helpful and responsive to our questions […] I felt supported and […] valued” (Second year living at home [196])), and proactive (“[An example of learning community is] when lecturers have acknowledged that we might be overwhelmed and have taken steps to reduce that anxiety” (First year living at home [234])). By listening to students, responding proactively to their queries, and acknowledging their difficulties, staff made students feel both valued and visible. Unsurprisingly, students who had been course representative or attended Staff-Student Forums (monthly meetings hosted by staff to facilitate conversations and resolve emergent issues between staff and students) appeared to have a heightened sense of this:

“Being part of [a staff-student forum] is a time when I have felt part of a learning community. Being given the opportunity to present opinions and thoughts to staff and feel that these are respected and taken into account when decisions are being made.” (Final year living with other students [170])

Being invited to give opinions or seek further support/advice and—importantly—these concerns being heard, acknowledged and understood was therefore a key element of the overall supportive approach. Again, this combines West and Williams’ (2017) “Access” and “Relationships” characteristics. Ready access to staff (“Access”) needed to come from within a mutually supportive network to develop a sense of interdependence (“Relationships”). It was this combination which fostered students’ feeling of being comfortable, respected, and, importantly, that they mattered.

Feeling “let in” to what may otherwise be perceived as an inaccessible academic world occupied by staff enabled students to feel included and accepted into a community which reached beyond their peers: “I suppose the closest [example of learning community] for me has been the discussions with my personal tutor, about study and their work in general” (Second year living alone [80]). The interaction referred to by this respondent reveals an intersection between the academic and the personal in which they realise “how collaborative the world of academia is and how specialised each expert is in their own field” and shifts the experience from being purely instructive, to “eye-opening” and revelatory (Second year living alone [80]). The student is enthused by being given access to academic insights which reach beyond educational content: they hear the lived experience of *being* an academic, rather than the knowledge required to become one. As such, this interaction feels more intimate and special, and adds to a sense of shared space and community. In general, having the opportunity to speak with staff on a 1–1 basis or in small group settings was seen as helpful: “Personally I [feel like there’s a learning community] when there are live seminars/Q&As that I engage with but sometimes other students don’t so it feels more like a 1:1” (Second year student living at home [56]).

Being granted access to events such as Research Seminars, which may typically only be offered to staff and postgraduate students, cemented students’ sense of importance and builds on this idea of exclusivity. One respondent noted, “There is a new [research] seminar series […] the seminar is packed with my lecturers and a few peers and provides a sense of community” (Final year living with other students [29]), whereas another indicated they “really appreciated how [academic staff member] has involved students in the extra seminars, by sending us the links” (First year living at home [114]). Note the primary reason Research Seminars were cited as examples of learning community here was not due to the academic content of the sessions, but the connection with staff and sense of inclusion such sessions granted. In the former extract, the respondent foregrounds and emphasises the seminar being “packed with my lecturers and a few peers”, with the session content placed in an auxiliary position. Similarly, in the latter extract, the student expresses appreciation merely at being invited and involved by a particular staff member; the seminar content is given no credence at all.

Being “packed” in with staff in a prior-to exclusive space connects with the idea of being “surrounded” alluded to by several students in their definitions of learning community “a supported, inclusive environment surrounded by other people” (Second year living with other students [213]); “Feeling surrounded and supported by peers and staff” (Final year living with other students [235]). For one respondent, this idea was taken even further when they related it to “sort of family” (Final year living in student halls [6]). This space must feel “supported” and “inclusive”, yet also exclusive in that it relates to a particular group of people—those “related to the learning of the school/university” and that feel part of the “sort of ‘family’” within that space. Simply sharing space and time with staff and feeling included in otherwise exclusive events and interactions thus enhances a community feel.

As several students noted, however, there is a need for balance. Not all felt able or wanted to be part of a wider learning community. For one respondent, this was related to there not being “much of a community among students overall”, and certainly not one they were “interested in being a part of” (Second year living at home [34]). However, this same respondent noted, “I do feel supported by staff as much as I need to be” (Second year living at home [34]). For another, the thought of being involved in a learning community was onerous as they “have limited resources in terms of my mental energy” and “would rather focus on getting things done that need to be” (Second year living alone [80]). As with the previous respondent, there was no sense of loss attached to this lack of learning community; indeed, the student in question was well “aware that the support and help is there if I need it” (Second year living alone [80]). For others, the very thought of developing a learning community was stressful given the pressures of the pandemic and they simply “could not care less” (Final year living with other students [248]). This respondent felt they were “too busy trying to keep my head above water and trying to stay on top of work” (Final year living with other students [248]) to care about making any further friends or building community*.*

Acceptance and a non-judgemental approach must therefore extend to those who do not wish to engage in a learning community beyond their academic work. Note, however, that orientations here are focused on either assumed extra/additional activities, which respondents feel unable to take part in due to other current pressures (“I would rather focus on getting things done that need to be”), or a lack of interest in the existing learning community (“I do not feel there’s much of a community […] that I am interested in being a part of”), rather than learning community per se. This may be more indicative of objection to the types of initiative taken to date and/or current dynamics within the School, rather than objection to inclusion overall.

### Feeling together: mutuality and the shared experience

As several respondents emphasised, working *with* staff rather than staff working *for* them was key to developing a sense of togetherness. This not only enabled mutual learning and development—“Having opportunities to work with other students and with staff is important to a learning community as it allows us to learn from each other” (Final year living with other students [170])—but, by “staff listening and working with us”, boosted the university experience, such that they could “get the most out of uni” (First year living in student halls [243]). The acknowledgement that learning is a mutual process shared by staff and students, was expanded upon further by another respondent, who noted: “a learning community is a group where everyone, even the people with higher education degrees, are still learning” (First year living at home [114]).

The use of “admitting” and “acknowledging” here suggests the ongoing nature of staff learning is often hidden, perhaps alluding to more traditional, passive forms of learning in which students are the receivers of knowledge passed on by masters of the subject. Against this model, in a learning community, staff were fellow *agents* of learning, regardless of academic achievement to date—“even” those who had earnt the scholarly title of “doctor” hadn’t “stopped learning”. Such concessions meant academic staff were not on an altogether different plane of existence to students but were continuing to “contribute to” knowledge and were “still learning” alongside them. As such, academic staff came to share the same plane of *experience* as those they were teaching: although at different stages of academic expertise, they shared the reciprocal experience of learning alongside one another. As in West and Williams’ (2017) “Vision” characteristic, they were “progressing as a community towards the same end” (p. 1576), i.e. academic advancement. This sense of togetherness was cemented by a non-judgemental attitude towards those at an earlier stage in the process where “no one will be treated as inferior for asking ‘stupid questions’” (First year living at home [114]). By sharing the plane of academic experience in this way, students may feel more accepted, staff may become more relatable, and academic success potentially feels more achievable.

Similarly, knowing peers within a learning community not only shared academic studies, interests and passions (“sharing a common interest in an academic discipline” (First year living in student halls [104]), “share the same interest in and passion for a subject” (Second year living with other students [176])), but also struggles and difficulties, was integral to a sense of togetherness. For some, knowing peers were available to “help each other when we don’t understand something” (First year living in student halls [51]) and have mutual experiences when it came to difficult material (“[a learning community is] when everyone on a group chat is complaining about the same lecture that I was struggling with” (First year living in student halls [252])) provided a sense of validation and reassurance. As one respondent put it, when others admitted “they didn’t understand the content” it felt “like we were all in the same boat”, helping to assuage concerns: “I was calm about it then” (Second year living alone [113]). Realising they were “all in the same boat” created not only a sense of shared experience, but also of reciprocity, allowing students to “help each other” (Second year living alone [113]). This aligns with West and Williams’ (2017) “Vision” and “Function” characteristics of learning community. On a most basic level, community was achieved here simply by students working on the same tasks, thus achieving functional cohesion. However, they also shared a sense of purpose (“Vision”)—working towards the same goal helped them to “think” like a community (West & Williams, 2017, p. 1575).

For those who did not have access to direct contact with others studying the same modules (both online and in-person), the lack of reciprocal problem sharing was clearly problematic. One respondent noted they would have really benefitted from “some sort of place to ask questions to other students to see if they are struggling with the same things as me” (Final year living with other students [284]). Knowing whether others were also “struggling” was given equal footing to sharing “tips on finding and researching information” (Final year living with other students [284]) by this respondent, suggesting that having the opportunity to share the *emotional* experience of studying the module was as important as the sharing of knowledge and information about the content.

For some students, however, there was a sense of disillusionment from the learning community which was connected to a perceived lack of commonality with others within the School. For some this was due to the department not being their parent school, being a mature student, living outside of student accommodation, or coming from a less traditional non-HE background. For example, one student noted “It is harder for me as I’m a mature student and I live at home” (First year living at home [223]), whereas another noted they had felt “isolated from other students” throughout their time at university, which they put down to coming “from a background where I was lucky to get into university, whereas my peers saw university as a natural progression” (Final year student living at home [98]). For these respondents, a sense of difference existed prior to the pandemic, yet the move online may have exacerbated this further due to the reduction in opportunities to interact with others: “being able to attend University would make me feel more as a part of community” (First year living at home [223]). Strikingly, one respondent felt their case did not even “really count” (First year living in student halls [166]) due to this not being their parent department, indicating a clear sense of disconnection from the School. Perceived exclusion in these cases was both implicit and explicit. For some, a lack of belonging was linked to a difference in experience and perceptions of HE prior to university (“I come from a background where I was lucky to get into university” (Final year student living at home [98]))*,* whereas others had concrete examples of being excluded. For example, one respondent felt they were not receiving all of the correct communications—“I feel like I’m not receiving all the information I should” (Final year living with other students [24])—whilst another was not able to participate in key aspects of the course due to being a mature student: “I am actually worried that I cannot make enough credits…due to the age limitations” (First year living at home [114]). This experience of difference and lack of belonging inevitably led to a sense of frustration and isolation in these respondents.

Overall, a sense of mutuality and shared experience appeared crucial to the underpinning of learning communities, which closely echoes the “Vision” characteristic outlined by West and Williams’ (2017) framework. As some respondents indicated, too much focus on the individual and working in isolation can come at a loss of community feeling; one respondent reported “the reality of uni is that it’s quite individual and most of the learning is done individually rather than in a community” (Final year living with other students [239]). As West and Williams (2017) stress, learning communities defined by a boundary of shared “Vision” may prompt a shift from the individual to broader, shared, common senses of purpose. Without a clear sense of mutuality and togetherness, the concept of learning community may become no more than a meaningless “*vague term*” (Final year living with other students [239]).

## Discussion

Overall, this study aimed to investigate how undergraduate students define, understand, and relate to the somewhat slippery and ill-defined concept of learning community in Higher Education. This was investigated in the context of remote online teaching prompted by the COVID-19 pandemic. Our reflexive thematic analysis of students’ first-hand accounts generated three dominant themes, which spoke to students’ desire to feel “connected”, “included”, and “together”. Students’ construction of learning community online as a collective, connected, mutual experience (with both staff and fellow students) allows a more comprehensive discussion about the value of building communities in the pedagogical literature. This analysis has thus revealed the more latent and nuanced content of learning community online in a way that champions the student voice(s).

The themes generated generally align with West and Williams’ (2017) model of the defining characteristics of learning communities pre-COVID-19. For example, their concept of a shared “Vision” (which incorporates shared goals and missions) and “Function” (which encompasses shared actions) spoke directly to our third theme of mutuality and the shared experience (or “feeling together”), whereas the concepts of “Relationships” (which encompasses sense of belonging and trust) and “Access” (which incorporates shared space and time) converged in our themes of “feeling connected” and “feeling included”. Our analysis therefore extends West and Williams’ (2017) initial framework by identifying how these boundaries of learning communities are forged, navigated, and maintained in an online context. This suggests that while online contexts disrupt the “spatial/geographic” communities that may foster a learning community in traditional settings, this sense of a shared vision and relationship building can be afforded online through curating a feeling of togetherness, mutuality, and sharing. This also aligns with Rovai et al.’s (2004) idea that learning communities comprise of people who have “ready access” to one another, which can be facilitated without the need for shared geographic space. More broadly, this echoes Ando’s (2021) sociocultural discussion of teaching and learning during COVID-19, who notes “learning is often a collaborative and human-dependent process” (p. 442). We appreciate the acknowledgement of the collaborative, inter-dependent nature of teaching and learning, and welcome more perspectives on learning communities that reflect these values.

Our study has also demonstrated that whilst pedagogic concepts such as “sense of belonging” and retention are inextricably tied to learning communities (e.g. Johnson, Madill, et al., 2020; Johnson, Sprowles, et al., 2020; Peacock & Cowan, 2019), there may be more complex drivers of this relationship. For example, some students conceptualise a learning community as being related to visibility and accessibility of academic teaching staff, which may affect sense of belonging, and others value the opportunity to share their experiences with fellow students. This inherently aligns with the “Vision”, “Relationships”, and “Access” characteristics of West and Williams’ (2017) framework, because it outlines the power of students sharing experiences and missions, with the affordance of reciprocity, quality time and ready access. Moreover, it is important to note the subset of students who consciously (and happily) elected to “opt out” of learning community online initiatives, which is more akin to the “functional” aspect of learning communities identified in the literature (West & Williams, 2017). This may mirror the escalating work, social, and academic demands on contemporary undergraduate students, and prompts us, as educators, to consider whether opting out of pedagogical and/or social offerings is problematic in and of itself. This may also reflect differences in instructional design of online teaching; that is, some modalities of teaching (e.g. asynchronous pre-recorded lectures) may inspire less engagement in online learning communities compared with other pedagogical online designs (e.g. synchronous discussions). There has been relevant discussion surrounding “performative engagement”, in which students “perform” the act of engagement to satisfy perceived expectations of academics (e.g. see Macfarlane, 2015). Thus, future work should now more explicitly consider whether active participation in learning community online may constitute a form of “performative engagement”, rather than actual, meaningful engagement, and the extent to which this matters for the student experience.

Finally, with these findings in mind, we invite follow-up studies that extend these enquiries with different groups; for example, by examining how these needs of feeling connected, included, and together may also translate to faculty online learning communities. This would enable us to further qualify the inconsistencies with institutional definitions and interpretations of learning community. In turn, this would provide us with the opportunity to recommend alternative measures (that directly link to student needs) for collation by institutions to truly understand their student satisfaction in learning online. A recent study by Bailey et al. (2021) qualitatively examined faculty’s engagement in learning community meetings and observed benefits such as a space for sharing concerns and experiences, bridging the gap between research and scholarship, fostering a sense of belonging and collegiality among faculty. There may also be value in investigating how different groups of students, staff, and other stakeholders experience learning communities differently, through a lens of inclusion and representation. Finally, in terms of wider policy implications, we now urge educators and scholars to critically consider how the terminology used in metrics across the sector (e.g. in this case, the NSS) may contain wording that staff and students may not share a unified definition of. This paper has ultimately highlighted the vast nuances that exist within perspectives of pedagogic terminology, focusing here on learning community. Thus, policy makers should aim to appreciate such perspectives by either (a) clarifying concepts clearly or (b) centring student voice in Higher Education metrics.

## Data Availability

Data for this study can be openly accessed here: https://osf.io/zw94s/.

## Footnotes

1 Note that the analytical process was recursive in that we did not follow the phases in a linear fashion but moved back and forth between them as needed (Braun & Clarke, 2006, p.86).
